# Hepatitis A Surveillance and Vaccine Use in China From 1990 Through 2007

**DOI:** 10.2188/jea.JE20080087

**Published:** 2009-07-05

**Authors:** Fuqiang Cui, Stephen C Hadler, Hui Zheng, Fuzhen Wang, Wu Zhenhua, Hu Yuansheng, Xiaohong Gong, Yuansheng Chen, Xiaofeng Liang

**Affiliations:** 1 Chinese Center for Disease Control and Prevention, Beijing, China; 2 WHO representative office in China, Beijing, China

**Keywords:** hepatitis A, epidemiology, surveillance, vaccine

## Abstract

**Background:**

Hepatitis A vaccines have been highly effective in preventing hepatitis A. To investigate the epidemiology of hepatitis A in China after hepatitis A vaccine became available, we reviewed reported cases of hepatitis A and the use of hepatitis A vaccine in China during the period from 1990 through 2007.

**Methods:**

Data from the National Notifiable Disease Reporting System from 1990 to 2007 and the Emergency Events Reporting System from 2004 to 2007 were reviewed and epidemiologic characteristics analyzed. Hepatitis A vaccine distribution between 1992 and 2007 was also reviewed.

**Results:**

The incidence of hepatitis A has declined by 90% since 1990, from 56 to 5.9 per 10^5^/year. Declines in age-specific incidence were seen in all age groups, most dramatically among children younger than 10 years. Disease incidence still varies substantially: poorer western provinces have had the highest incidences since 2000. In high-incidence provinces, children younger than 10 years continue to have a high disease incidence. Only 50% of cases were laboratory-confirmed, and only 3% occurred in reported local outbreaks. Over 156 million doses of hepatitis A vaccine have been distributed since 1992, and use has continued to increase since 2003.

**Conclusions:**

Incidence of hepatitis A has decreased in all age groups, likely due to changing socioeconomic conditions and increasing hepatitis A vaccine use. Nevertheless, western populations remain at high risk, with transmission predominantly occurring among children. The epidemiology of hepatitis A transmission is not well understood. Improved surveillance with better laboratory confirmation is needed to monitor the impact of universal hepatitis A vaccination of young children; this strategy began to be implemented in 2008.

## INTRODUCTION

Hepatitis A is an acute, usually self-limited infection of the liver caused by the hepatitis A virus (HAV). Transmission occurs primarily through the fecal–oral route, and is closely associated with poor sanitary conditions.^1^^,^^2^ In most developing countries, hepatitis A is highly endemic and infection usually occurs in early childhood. Among children younger than 5 years, 70% of those infected are asymptomatic; therefore, in high prevalence areas, symptomatic cases are uncommon.^1^ Nevertheless, hepatitis A can result in a high disease burden, and is still considered a public health problem in many countries. During the 1980s and 1990s, improved sanitation resulted in an increase in the average age of hepatitis A onset in many countries, but most cases still occur in populations living in poor sanitary conditions.^2^^–^^5^

Hepatitis A vaccines are highly effective in preventing both clinical hepatitis and in reducing disease spread. Vaccination against hepatitis A has resulted in robust health and economic benefits in Argentina.^6^ Recent evidence from Israel, Alaska, the United States, and northern Australia indicates that vaccination of young children has markedly decreased hepatitis A incidence among all age groups.^7^^–^^9^

In China, hepatitis cases have been reported as part of the National Notifiable Disease Reporting System (NNDRS) since 1959, and have been reported separately by virus type since 1990. NNDRS is a hospital-based, passive national surveillance system that has included all county hospitals in 31 provinces since 1959. Before 2003, cases were reported monthly to county Centers for Disease Control and Prevention (CDCs) via hardcopy (case and hardcopy-based NNDRS), and through prefecture and provincial CDCs to national authorities. Case information included age, sex, location of residence, and date of onset. In 2004, the China CDC revised the reporting system, establishing an online electronic version (case and computer-based). After diagnosis, cases are reported via the Internet from all county and most township-level hospitals. In addition, all outbreaks (defined as more than 5 cases in 1 village within 1 week) must be reported immediately to the Emergency Events Reporting System (EERS) by the county CDC. The Emergency Events Reporting System was established in 2004 as an online electronic system accessible by county, prefectural, provincial, and national CDCs through the Internet. The Emergency Events Reporting System provides information on the basic epidemiologic characteristic of outbreaks. The largest documented hepatitis A outbreak in the world occurred in Shanghai in 1988, with more than 300 000 persons infected, and with estimated direct costs of $58 million and indirect costs of $64 million.^10^

In China, hepatitis A is a public health concern, as over 60 000 cases are currently reported annually. The most common recognized modes of transmission of outbreaks in China are ingestion of food and water^11^^,^^12^ contaminated by hepatitis A virus, as is the case in other countries.^13^ However, many individuals are likely infected through close personal contact with an infected person.^14^ An effective live, attenuated hepatitis A vaccine has been available for private use (class 2) since 1992,^15^^,^^16^ and inactivated vaccines became available in 2002.^17^ These vaccines have been used increasingly since 2000 to prevent hepatitis A, primarily in school-aged children.

This report summarizes the status of reported cases of hepatitis A and hepatitis A vaccine use in China during the period from 1990 through 2007. In addition, the implications for future control with hepatitis A vaccination are discussed.

## METHODS

This is a descriptive epidemiologic study that reviewed national surveillance data and vaccine distribution data in China. We reviewed incidence data on reported hepatitis A from 1990 to 2007 from the NNDRS, in which a reportable case is defined as an acute illness with discrete onset of symptoms with jaundice and/or elevated serum aminotransferase levels (suspected case). A case is considered confirmed if a person tests positive for the IgM antibody to hepatitis A virus or is epidemiologically linked with a lab-confirmed case. Information compiled for each case since 2004 includes whether the case has been laboratory confirmed, although it does not designate what type of test was used to confirm the diagnosis.

In addition, since 2004, China’s Emergency Events Reporting System has collected information on all outbreaks of communicable diseases; outbreaks are defined as 5 or more cases in the same village within a 1-month period. Information on each outbreak includes number of cases, date of outbreak cases, primary location, and the age groups affected.

NNDRS data on hepatitis A from 1990 to 2003 and 2004 to 2007 were analyzed. This was a period of national transition from high to low hepatitis A incidence. Case numbers and disease incidence were examined by year, age, province, and provincial grouping; disease seasonality, case sex, occupational group, and frequency of laboratory confirmation were also examined for the period from 2004 to 2007. Age-specific incidence was examined nationwide and in provinces, and grouped according to incidence. We defined the relative incidence for each province—according to the average incidence rate between 2003 and 2007—as high (>8/10^5^/yr), intermediate (4–8/10^5^/yr), or low (<4/10^5^/yr). Incidence in 10 (mainly western) provinces was classified as high; 10 provinces had intermediate incidences, and 11 (mainly eastern and central provinces) had low incidences.**High incidence: Xinjiang, Xizang (Tibet), Qinghai, Gansu, Ningxia, Sichuan, Yunnan, Guizhou, Chongqing, Hainan. Intermediate incidence: Jiangxi, Henan, Liaoning, Shaanxi, Zhejiang, Neimenggu, Fujian, Jilin, Hubei, Guangxi. Low incidence: Anhui, Shanxi, Jiangsu, Heilongjiang, Shanghai, Hunan, Beijing, Hebei, Shandong, Guangdong, Tianjin.
Within each province, an epidemic was defined as a 50% or greater increase in incidence, as compared to the lowest incidence of the previous 3 years. Incidence rates were calculated based on the most recent National Statistical Bureau census data, those of 2000. The 2007 Chinese population is estimated to be 1.3 billion, with a birth rate of 1.209% (birth cohort of 15.7 million).^18^

Hepatitis A outbreaks reported to the Emergency Events Reporting System from 2004 to 2007 were reviewed, including the number of outbreaks, the number of cases in each outbreak, and outbreak location. Hepatitis A vaccine distribution data for the period from 1992 through 2007 were provided by the Chinese domestic companies that produce most of the childhood vaccines used in China. However, these data do not include information on vaccine distributed by non-Chinese vaccine companies.

## RESULTS

### National trends in hepatitis A incidence

The reported incidence of hepatitis A in China has decreased rapidly since 1990, when hepatitis A first became reportable as a distinct disease: from 56/100 000 in 1991 (584 353 cases) to 5.9/100 000 in 2007 (Table 1).^18^ In a recent 4-year period (2004–2007), between 68 667 and 93 587 cases of hepatitis A have been reported through the NNDRS; incidence in these years was 7.2/100 000, 5.6/100 000, 5.4/100 000, and 5.9/100 000, respectively. During this period, there were no significant national differences in incidence by month or season; however, incidence was higher in western provinces, and modest seasonality (peaks in spring or summer) was seen in several western provinces. In addition, there was a seasonal increase in hepatitis A outbreaks in April and September each year.

**Table 1.** Annual incidence of hepatitis A in China: 1990–2007 (1/10 000)

Year	No. of Cases	Incidence	Year	No. of Cases	Incidence
	
1990	584 353	52.6	1999	211 501	17.0
1991	637 717	55.7	2000	134 094	10.8
1992	602 591	52.1	2001	122 896	9.7
1993	457 895	39.5	2002	108 490	8.1
1994	353 388	30.3	2003	97 147	7.3
1995	254 242	21.5	2004	93 585	7.3
1996	238 331	19.9	2005	73 349	5.8
1997	226 599	18.8	2006	68 667	5.2
1998	200 337	16.0	2007	77 135	5.9

Source: NNDRS

### Age distribution of hepatitis A incidence

**Figure 1.**
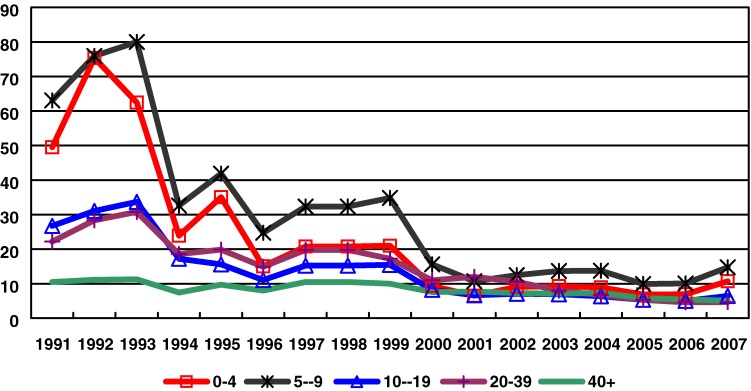
Incidence of hepatitis A by age group: 1991–2007. Source: NNDRS.

Data from NNDRS from the early 1990s show that the highest incidence was in children aged <10 years, followed by persons aged 10–19 years and 20–39 years (Figure 1). In the mid-1990s, incidence decreased rapidly and then remained relatively stable from 2002 to 2007. The decline in incidence was seen in all age groups, but most dramatically among children younger than 10 years, among whom incidence declined from between 60–76/10^5^/yr to 10/10^5^/yr. Currently, hepatitis A incidence remains highest among children younger than 10 years (10–15/10^5^/yr), and is only slightly lower among older age groups. Among hepatitis A cases reported between 2004 and 2007, 10% are children under 5 years, 17% are children aged 5–9 years, and 19% are persons aged 10–19 years.

### Regional variation of hepatitis A incidence

**Figure 2.**
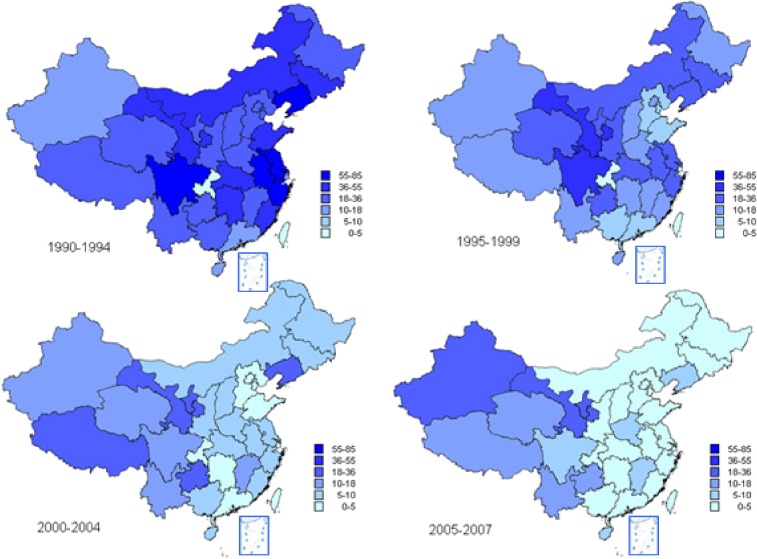
Incidence of hepatitis A by province: 1990–2007. Source: NNDRS.

**Figure 3.**
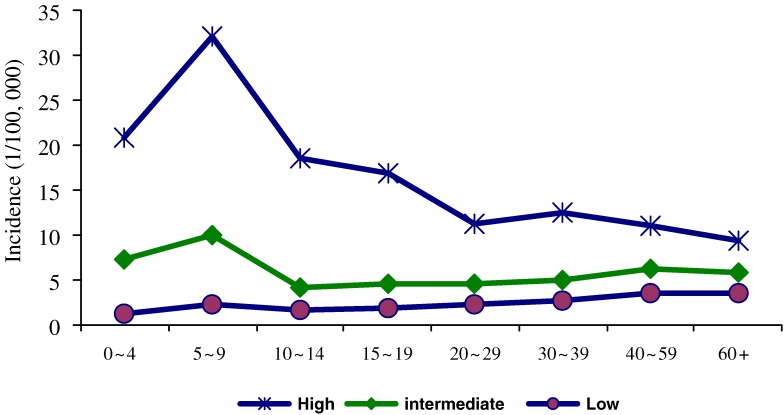
Age-specific incidence of hepatitis A in areas of high, intermediate, and low incidence: 2004–2007. Source: NNDRS.

During 1990 to 1994, the eastern provinces of China reported higher incidence than those in western China, but incidence in eastern China then declined more dramatically than in western China. From 2005 to 2007, the period of lowest national incidence, the observed incidence in western provinces was substantially higher than in eastern provinces (Figure 2). In provinces with high incidences (>8/10^5^/yr between 2003 and 2007), the age-specific incidence during 2004–2007 was highest in children—reaching 20–30/10^5^/yr for children younger than 10 years—and declined with increasing age to 10–15/10^5^/yr among adults. For provinces with intermediate incidences, incidence was also higher in children younger than 10 years, but similar among older age groups. In contrast, among provinces with low incidences, adults had a higher incidence than did children (Figure 3). Among 10 high-incidence provinces, 9 had periodic epidemics every 4–8 years, including at least 1 epidemic between 2000 and 2007; however, among 21 provinces with intermediate or low incidences, only 4 had an epidemic during this period.

### Distribution of hepatitis A by sex and occupation

Among reported cases in 2004 to 2007, a total of 206 405 were male and 106 330 were female (66% and 34%, respectively)—a male-to-female case ratio of 2:1. During this period, with respect to occupation, 118 214 cases were farmers, 75 369 were students, 28 459 were preschoolers, and 90 693 were classified as other, accounting for 37.8%, 24.1%, 9.1%, and 29% of cases, respectively.

### Reported outbreaks of hepatitis A in China

Although disease incidence has decreased, local outbreaks remained common, particularly in western provinces. Among hepatitis A cases reported between 2004 and 2007, a total of 9197 (2.9%) were from 154 reported outbreaks (Table 2). The outbreaks occurred mostly in rural primary and middle schools with inadequate water supply, or resulted from suspected consumption of contaminated cold food in western provinces. Among outbreaks that occurred in 2004 to 2007, 49.0% were in primary, middle, and high schools. Of the 154 reported outbreaks, 76 occurred in high-incidence provinces, 44 in intermediate-incidence provinces, and 34 in low-incidence provinces.

**Table 2.** Laboratory-confirmed hepatitis A cases and number of cases in outbreaks from 2004–2007

	Total	Confirmed by lab		Outbreaks		
	
	Cases	Cases	%	No.	Cases	%
2004	93 587	46 221	49.4	26	2291	2.4
2005	73 349	34 196	46.6	33	2061	2.8
2006	68 667	35 735	52	43	1826	2.7
2007	77 135	45 786	59.4	52	3019	3.9
Total	312 738	161 938	51.8	154	9197	2.9

Sources: NNDRS (Total and Laboratory-Confirmed cases); EERS (Outbreaks).

### Laboratory diagnosis of hepatitis A

Among the hepatitis A cases in reported in 2004 to 2007, 48.2% were clinically confirmed and 51.8% were confirmed on the basis of laboratory findings. The proportion of cases that were laboratory-confirmed increased from between 47–49% between 2004 and 2005 to 59% in 2007 (Table 2).

### Hepatitis A vaccine use in China

Two types of vaccine are available in China: live, attenuated hepatitis A vaccine, which was licensed in 1992, and inactivated vaccine, available since 2002. Both are produced domestically. Before 2008, hepatitis A vaccine was a class 2 vaccine, ie, not routinely recommended for childhood vaccination, and was available to persons willing to pay for vaccination. Live, attenuated hepatitis A vaccine is given as a single dose to children older than 18 months; inactivated hepatitis A vaccine is given in a 2-dose series. The Chinese scientific literature shows that both vaccines are highly effective against HAV in China.^15^^–^^17^^,^^19^

**Figure 4.**
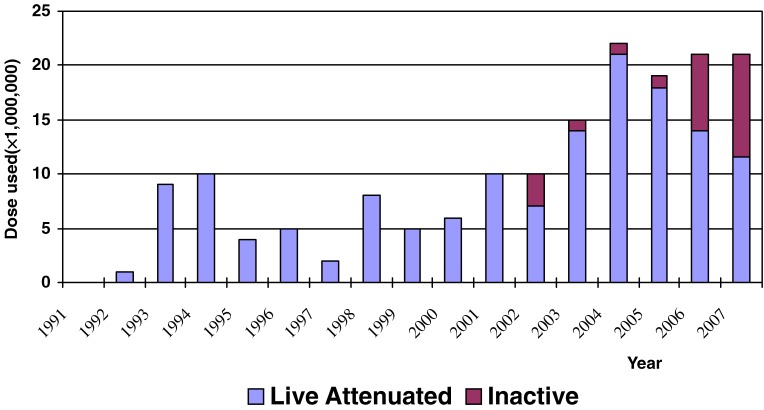
Hepatitis A vaccine distributed by year: 1992–2007. Source: China Domestic Biologic Companies

Between 1992 and 2007, 156 million doses of hepatitis A vaccine were distributed, mostly for school-aged children (Figure 4). The total vaccine distributed from 2004 through 2007 (18 to 22 million doses each year) was sufficient to vaccinate approximately 1 child birth cohort each year (approximately 16 million children).

## DISCUSSION

Disease surveillance data reveal a dramatic decrease in reported hepatitis A cases in China during a recent 17-year period. Decreases were observed in all age groups and were greater in the eastern provinces of China.

Although the causes for the marked reduction in hepatitis A incidence in China cannot be determined by this study, we suspect that several factors are important. First, with continuing economic development, the quality of water, food, and sanitation has substantially improved. It is therefore likely that fewer people became infected through water and food, and that transmission through close contact was also reduced due to improved sanitation and life style. We believe that these factors contributed to the rapid decline in HAV risk in China during the 1990s.^20^

Second, hepatitis A vaccination may now be playing a substantial role. The cumulative output of hepatitis A vaccine from Chinese manufacturers increased from 2.9 million doses in 1992 to 156 million doses through 2007.^21^ Although no age- or province-specific immunization coverage data on hepatitis A are available, hepatitis A vaccine has been given mostly to school-aged children; the number of children in each school class is approximately equal to the number of persons who can be vaccinated each year with the amount of vaccine now available.^17^ In addition, it is likely that hepatitis A vaccine has been used mainly in the wealthier eastern provinces, where parents can more easily afford to vaccinate their children. Studies of population vaccination with hepatitis A vaccine have highlighted the importance of herd immunity, which becomes apparent with coverage of 66% or higher.^8^^,^^22^ Thus, hepatitis A vaccine may have provided significant protection to children, and to the entire population as well.

Thus, in the well-developed eastern and urban regions, a booming economy, improvements in sanitation and life style, and increasing vaccination rates have likely played major roles in risk reduction. Indeed, populations in these regions had lower prevalences of HAV antibodies in a 1992–1995 national serosurvey.^23^ Furthermore, with additional reductions in disease transmission, fewer adults will develop natural infection, and a higher proportion of the older adult population will be susceptible. If vaccination is not continued, there may be increased risks of disease and outbreaks in adults, among whom disease progression is more severe. Therefore, the current vaccination program will play an increasingly important role in protecting the population in the future.

Due to differences in sanitary environments within China, there were no significant national differences in incidence by month or season; however, in western areas with lower economic and sanitary conditions, incidence was higher in all age groups. In addition, there was a seasonal increase in hepatitis A outbreaks in April and September every year in those provinces, possibly due to school-related outbreaks, which were commonly reported but accounted for only 3% of cases. In rural areas, outbreaks mostly occurred in rural primary and middle schools, which have inadequately protected drinking water and where transmission through close contact is more likely.^12^

There were considerably more male cases than female cases, perhaps due to greater health care seeking among males and to more exposures in the workplace, where hygiene and access to clean food and water may be worse. Although preschoolers and school-aged students made up only 33% of cases, incidences were higher in these age groups and were likely highest in preschoolers, since this age group is more likely to be asymptomatic after infection. Among adults, distribution of disease in farmers and other groups was consistent with occupations in China overall.

Despite the steadily improving economy and decreasing incidence of hepatitis A, some populations remain at high risk and continue to experience periodic epidemics and frequent smaller outbreaks. In these areas, which are mainly western and rural, disease risk continues to be highest in young children. However, risk factors remain poorly defined and further study is needed to examine the changing risks for HAV in China.

Many challenges remain before we will be able to clearly define the burden and cost of hepatitis A in China. In 2004–2007, only 51.8% of cases were laboratory-confirmed, because of the high cost of testing and the lack of laboratory facilities in poorer townships and villages. The risk factors for hepatitis A and the overall cost associated with hepatitis A in China are not well known. Efforts are needed to increase the systematic use of confirmatory laboratory tests at peripheral health centers, to define the risk factors for disease in populations at high risk, and to determine the cost of the disease and the cost effectiveness of vaccination for at-risk populations.

A key limitation of the present study is that it is a descriptive study based on surveillance data and reports of vaccine distribution. These data cannot directly evaluate the causes of decreased incidence, such as improvements in sanitary conditions and increased use of hepatitis A vaccination. Furthermore, the quality of the reported data, particularly the accuracy of the diagnosis and laboratory testing, cannot be verified from the available data.

In order to further reduce the transmission of HAV, China has integrated hepatitis A vaccination into the routine child immunization program in 2008, and provides vaccine for children beginning at the age of 18 months. Initially, all children in 2 high-incidence provinces—Xinjiang and Yunnan—will be vaccinated; the remaining provinces will initially be able to vaccinate only 40% of children. Hepatitis A vaccine is expected to be available for all children by 2010. This approach to disease control is based on the strategies for hepatitis A prevention used for high-risk populations in more developed countries, which have demonstrated how vaccination of young children has a dramatic impact on hepatitis A incidence in all age groups.^7^^–^^9^ Furthermore, to better measure the impact of immunization and refine future immunization strategies, China plans to enhance laboratory-based surveillance of hepatitis A and to evaluate the economic cost of hepatitis A. By combining universal vaccination with stronger surveillance, China expects to continue to reduce the risk of hepatitis A in the entire population.

## References

[1] WHO, Weekly Epidemiological Record. Hepatitis A vaccines, WHO position paper, 2000, 75, 37–44.

[2] Hadler SC. Global impact of hepatitis A virus infection—Changing patterns. In Hollinger FB, Lemon SM, Margolis H (eds) Viral Hepatitis And Liver Disease. Williams and Wilkens, Baltimore, MD 1991.

[3] Barzaga BN Hepatitis A shifting epidemiology in South-east Asia and China . Vaccine. 2000;18 S1:S61–4 10.1016/S0264-410X(99)00467-3 10683551

[4] Jacobsen KH , Koopman JS The effects of socioeconomic development on worldwide hepatitis A virus seroprevalence patterns . Int J Epidemiol. 2005 Jun;34(3):600–9 Epub 2005 Apr 14 10.1093/ije/dyi062 15831565

[5] Mausezahl D , Cheng F , Zhang SQ , Tanner M Hepatitis A in a Chinese urban population: the spectrum of social and behavioral risk factors . Int J Epidemiol. 1996;25:1271–9 10.1093/ije/25.6.1271 9027535

[6] Lopez E , Debbag R , Coudeville L , Baron-Papillon F , Armoni J The cost-effectiveness of universal vaccination of children against hepatitis A in Argentina: results of a dynamic health-economic analysis . J Gastroenterol. 2007 Feb;42(2):152–60 Epub 2007 Mar 12 10.1007/s00535-006-1984-x 17351805

[7] Dagan R , Leventhal A , Anis E , Slater P , Ashur Y , Shouval D Incidence of hepatitis A in Israel following universal immunization of toddlers . JAMA. 2005;294:202–10 10.1001/jama.294.2.202 16014594

[8] Wasley A , Samandari T , Bell B Incidence of hepatitis A in the United States in the era of vaccination . JAMA. 2005;294:194–201 10.1001/jama.294.2.194 16014593

[9] Chodick G , Green MS , Heymann AD , Rosenmann L , Shalev V The shifting epidemiology of hepatitis A following routine childhood immunization program in Israel . Prev Med. 2007 Nov;45(5):386–91 Epub 2007 May 21 10.1016/j.ypmed.2007.05.011 17599401

[10] Cooksley WG What did we learn from the Shanghai hepatitis A epidemic? J Viral Hepat. 2000 May;7 Suppl 1:1–3 10.1046/j.1365-2893.2000.00021.x 10870174

[11] Shen YG , Gu XJ , Zhou JH Protective effect of inactivated hepatitis A vaccine against the outbreak of hepatitis A in an open rural community . World J Gastroenterol. 2008;14(17):2771–5 10.3748/wjg.14.2771 18461664 PMC2709045

[12] Zhang LJ , Wang XJ , Bai JM , Fang G , Liu LG , Zhang Y , An outbreak of hepatitis A in recently vaccinated students from ice snacks made from contaminated well water . Epidemiol Infect. 2009 Mar;137(3):428–33 Epub 2009 Feb 9 10.1017/S0950268808001337 18817585

[13] Barrimah E , Salem KA , Gabal MS An outbreak of hepatitis A associated with treated waste water used for irrigation . J Egypt Public Health Assoc. 1999;74(3–4):227–39 17219868

[14] Zheng H , Lu Y , Wang FZ , Cui FQ Epidemiological Analysis on hepatitis A in China during 2004–2006 (English abstract) . Chinese Journal of Vaccines and Immunization. 2007;13(4):336–40

[15] Wang XY , Xu ZY , Ma JC , von Seidlein L , Zhang Y , Hao ZY , Long-term immunogenicity after single and booster dose of a live attenuated hepatitis A vaccine: results from 8-year follow-up . Vaccine. 2007;25(3):446–9 Epub 2006 Aug 17 10.1016/j.vaccine.2006.08.004 16949710

[16] Mao JS , Chai SA , Xie RY , Chen NL , Jiang Q , Zhu XZ , Further evaluation of the safety and protective efficacy of live attenuated hepatitis A vaccine (H2-strain) in humans . Vaccine. 1997;15(9):944–7 10.1016/S0264-410X(96)00304-0 9261939

[17] Zhuang FC , Qian W , Mao ZA , Gong YP , Jiang Q , Jiang LM , Persistent efficacy of live attenuated hepatitis A vaccine (H2-strain) after a mass vaccination program . Chin Med J (Engl). 2005;118(22):1851–6 16313838

[18] National Bureau of Statistical of China China Statistical Yearbook in 2007. China Statistic Press, Beijing, 2008.

[19] Jiang WP , Chen JT , Wang X , Wang YL , Yan Liu Y , Chen WY , Immunogenicity and safety of three consecutive lots of a new preservative-free inactivated hepatitis A vaccine (Healive®): A double-blind, randomized and controlled trial . Vaccine. 2008;26(18):2297–301 10.1016/j.vaccine.2007.11.008 18395305

[20] Wang ZS , Shepard DS , Zhu YC , Cash RA , Zhao RJ , Zhu ZX , Reduction of enteric infectious disease in rural China by providing deep-well tap water . Bull World Health Organ. 1989;67(2):171–80 2501042 PMC2491231

[21] Xu ZY. Decline in Risk of HAV in China, a Country with Booming Economy & Changing Life Style, Abstract, Global Hepatitis A conference, December 12, 2007, Miami, FL, USA. P47.

[22] Averhoff F , Shapiro CN , Bell BP , Hyams I , Burd L , Deladisma A , Control of hepatitis A through routine vaccination of children . JAMA. 2001;286(23):2968–73 10.1001/jama.286.23.2968 11743837

[23] Dai ZC, Qi GM. Viral hepatitis in China: sero-epidemiological survey in Chinese population 1992–1995 (part one). Beijing: Scientific and Technical Documents Publishing House: 1999.

